# Effectiveness of a scenario-based, community-based intervention in containing COVID-19 in China

**DOI:** 10.3389/fpubh.2024.1449305

**Published:** 2024-11-27

**Authors:** Binghang Li, Yalin Zhou, Ting Zhang, Anning Ma, Wenhao Hao

**Affiliations:** ^1^Weifang People’s Hospital, Shandong Second Medical University, Weifang, China; ^2^School of Public Health, Shandong Second Medical University, Weifang, China; ^3^School of Population Medicine and Public Health, Chinese Academy of Medical Sciences and Peking Union Medical College, Beijing, China

**Keywords:** pandemic COVID-19, infectious disease dynamics modeling, non-pharmacological interventions, measure effects, scenario-based

## Abstract

**Background:**

Given the significant impact of the more than three-year-long COVID-19 pandemic on people’s health, social order, and economic performance, as well as the potential re-emergence of a new variant and the epidemic “Disease X,” it is crucial to examine its developmental trends and suggest countermeasures to address community epidemics of severe respiratory infectious diseases.

**Methods:**

The epidemiological characterization of various strains of COVID-19 was modeled using an improved Susceptible-Exposed-Infectious-Recovered (SEIR) model to simulate the infections of different strains of COVID-19 under different scenarios, taking as an example an urban area of a prefecture-level city in Shandong Province, China, with a resident population of 2 million. Scenarios 1–5 are scenario-based simulations the Omicron strain, and 6–8 simulate the original COVID-19 strain, with different parameters for each scenario. Scenarios 1 and 6 do not consider community NPIs and represent natural epidemic scenarios. Scenarios 2–4 assess the impact of different NPIs on the original COVID-19 strain. Scenarios 1–4 and 6–8 compare the effects of the same measures on different strains. Scenario 5 simulates the effects of implementing NPIs after an outbreak has spread widely. Compare scenarios 4 and 9 to analyze the effect of high grades versus dynamic clearing of NPIs. By analyzing the time at which the peak number of cases was reached and the maximum number of cases, we were able to calculate the effectiveness of urban community control measures (NPIs) and the impact of vaccination on disease trends. Based on our research into the degree of restriction of social activities in different levels of control areas during real-world epidemics, we categorized the NPIs into three levels, with controls becoming increasingly stringent from levels 1 to 3 as low-, medium-, and high-risk areas are, respectively, controlled.

**Results:**

In simulation scenarios 1–5 and 9, where the epidemic strain is Omicron and the susceptible population receives three doses of vaccine, it was found that the real-time peak number of cases in scenario 2, which implemented level 1 controls, was reduced by 18.19%, and in scenario 3, which implemented level 2 controls, it was reduced by 38.94%, compared with scenario 1, where no control measures were taken. Level 1 and level 2 controls do not block transmission but significantly reduce peak incidence and delay the peak time. In scenario 5, even with a high number of initial cases, the implementation of level 3 controls can still control the outbreak quickly, but it requires a longer period of time. However, Omicron has a low rate of severe illness, and the existing beds in City A could largely cope even if the control measures had not been implemented. Analyzing scenarios 4 and 9, level 3 community control and dynamic zeroing of the three zones were similarly successful in interrupting the spread of the epidemic. In simulation scenarios 6–8, where the prevalent strain was the original COVID-19 strain, only level 3 community control was able to rapidly extinguish the outbreak. Unchecked, the outbreak is severe, characterized by high peaks and substantial medical stress. Although level 2 controls reduced real-time incidence and peak new infections by 39.81 and 61.33%, and delayed the peaks by 55 and 52 days, respectively, the high rate of severe illnesses may still overwhelm the medical system.

**Conclusion:**

Control effects are related to the level, timing and virus characteristics. Level 3 and dynamic zeroing measures can interrupt community transmission in the early stages of an outbreak. During a pandemic, different NPIs must be implemented, considering the virus’s status and cost of control, and ensuring that medical resources are sufficient to maintain medical order.

## 1 Introduction

With the acceleration of globalization and the increasing frequency of population movements, the outbreak and spread of new pathogens have become a major challenge to global public health. Outbreaks of severe respiratory infectious diseases have caused a series of social problems, including deaths from illness and injury, the crowding out of medical resources, and the disruption of social order, especially in the case of the COVID-19 epidemic, which spread rapidly and had a wide range of effects. The COVID-19 pandemic has hit the global economy hard, with an average loss of $84 billion in global GDP in 2020. According to the World Bank’s Global Economic Prospects report released in June 2020, global GDP contracted by 5.2%, making the worst recession in decades (1). Although COVID-19 is currently under control, WHO Director General Tedros warns of a possible global outbreak of “Disease X,” suggesting that it is only a matter of time before new variants and epidemics emerge. At present, COVID-19 infection is most similar to “Disease X,” Therefore, a careful summary of the prevention measures for the new coronavirus epidemic and the experience of other countries will help us better deal with “Disease X” in the future.

In the face of the COVID-19 epidemic, China was the first country to adopt community control measures, showcasing its governance and institutional strengths and achieving remarkable results. The specific expressions of community control are mainly embodied in the blockade of infected areas, the prohibition of congregational activities, the establishment of a gridded and closed community control model that links “prevention, control and treatment,” and the implementation of medical control measures that are “graded, classified and triaged.” However, there is still a lack of systematic scientific proof regarding the intensity of control, the areas involved, the duration, etc. There are also issues such as excessive control, inadequate implementation of preventive and control measures, insufficient control through scientific preventive and control means, etc., which urgently need to be addressed based on scenario previews. The prevention and control of a COVID-19 pandemic provide an opportunity to study the epidemiological patterns and prevention and control strategies for influenza pandemics. Fortunately, the virulence and transmission characteristics of the virus can be estimated through the analysis of existing and previous outbreaks, enabling the use of mathematical methods to model disease transmission. Transmission dynamics models are widely used to analyze the epidemiological trends of infectious diseases. Based on simulations at different times, we can develop targeted prevention and control strategies and rationally allocate medical resources.

The uncertainty and inevitability of an influenza pandemic make prevention and control particularly crucial. The emergence of new influenza subtypes, along with the unpredictable timing and locations of their occurrences, poses challenges. There is even the possibility of concurrent influenza and COVID-19 pandemics. Therefore, exploring control measures is essential in the prevention and control of influenza pandemics. This paper compares COVID-19 infections and healthcare resource needs across different scenarios, aiming to provide quantitative evidence to support community control strategies for severe respiratory infections.

The remaining structure of this study is organized as follows: Section 2 provides a summary of related work and presents the research framework, which involves establishing a SEIR model and conducting simulation experiments to examine the transmission dynamics of severe respiratory infectious diseases. This section investigates the effects of model parameters and different epidemic control strategies on disease transmission. Section 3 presents the findings of numerical modeling, which quantifies the impact of relaxed and strict social control measures on disease transmission using empirical research data. The contributions and limitations of this study are discussed in Sections 4–6.

## 2 Mathematical modeling

Dynamics of COVID-19 outbreak transmission in A cities.

### 2.1 Data collection

The data required for community epidemiological risk assessment of severe respiratory infections are obtained through questionnaires, in addition to official statistics. In this study, a sample city, City A, a prefecture-level city in Shandong Province, was used for the empirical study. From March 10^th^ to March 28^th^, 2022, a total of 8 confirmed native cases and 86 native asymptomatic infections were reported in City A. This study is a cross-sectional survey of residents living in communities in City A that had been classified into different risk zones (low, medium, and high) during the epidemic. A total of 1,200 residents from 60 urban communities in the urban area of City A, spanning the three risk zones, were randomly selected using the simple random sampling method as the survey sites and respondents. Data on the frequency of residents’ travels during the epidemic were collected through the distribution of online questionnaires to ensure a wide distribution of respondents. The Revealed Preference (RP) method was used in this survey, focusing on collecting data about the choice behaviors that have actually occurred or been completed by the respondents. This method is particularly suitable for obtaining the real choices made by respondents under the current situation. The advantage of the RP survey is that it ensures the authenticity of the data and the model constructed is based on real measurements, enhancing the reliability of the model (2). A total of 1,190 valid questionnaires were collected from community residents across the 60 communities surveyed: low-risk zones (20 communities/456 people), medium-risk zones (29 communities/521 people), and high-risk zones (11 communities/213 people). This survey distribution provides valuable data to understand how the frequency of travel by community residents in different risk zones decreases due to varying levels of NPIs implementation.

The risk zone delineation and control program for the new coronavirus pneumonia epidemic, as outlined in the New Coronavirus Pneumonia Prevention and Control Programme (Ninth Edition) issued by the State Council’s Comprehensive Group of the Joint Prevention and Control Mechanism for Responding to the New Coronavirus Pneumonia Epidemic, were implemented. According to the program, Level 3 NPIs are implemented in the sealed Closure zone (high-risk zone), which is the area where cases and asymptomatic infected persons reside. Level 2 NPIs are implemented in the control zone (medium-risk zone), which is the area where cases and asymptomatic infected persons stay and move around, posing a risk of spreading the outbreak. The remaining counties (municipalities, districts, and flagships) where medium- and high-risk zones are located are classified as the precautionary zones (low-risk zones), and Level 1 NPIs are implemented there. The higher the risk, the more stringent the community NPIs adopted. The control measures for high-, medium- and low-risk areas are detailed in Table 1.

**Table 1:** Content of NPI measures at different levels.

NPI levels	Control measures
Level 1 (low-risk zones)	The implementation of “personal protection, avoiding gatherings” strengthens distancing measures. During this period, minimizing activities and staggering entry to indoor public places are encouraged. Flow control, temperature measurement, registration, and the wearing of masks are some of the precautionary measures in place.
Level 2 (medium-risk zones)	During the implementation of control measures, restrictions are placed on movement. People are advised to stay within their designated areas and there are staggered pick-up times and orderly procedures in place. Flow restrictions are implemented based on zoning, ensuring regulated movement of individuals.
Level 3 (high-risk zones)	During the period, the implementation of containment measures includes “door-to-door service without leaving home.”

### 2.2 SEIR segregated compartment modeling

We use the well-known SEIR model to simulate the propagation dynamics of COVID-19 in City A under different scenarios. Our modeling is based on pathogenic and epidemiological characteristics of COVID-19 and real-world parameters to assess the impact of community-based control measures (NPIs) on the intensity of COVID-19 pandemics and the protection provided by the vaccine against infection and disease severity.

#### 2.2.1 Modeling assumptions

1. After infection with COVID-19, there is a latency period during which the virus remains in the human body. According to relevant studies, both asymptomatically infected and latent individuals can transmit the virus (3). We assume that individuals infected during the incubation period are also somewhat infectious. However, in reality, there is a discount factor between individuals infected during the incubation period and those infected at the onset of the disease.
2. The total population in the study area remains constant. The impact of spillover cases and imported cases within the study area is not taken into account, as the proportion of migration out of and into the population over a given period of time is small. Additionally, the effects of births and natural deaths are not considered.
3. All individuals in a population have the potential to be infected, i.e., the population is universally susceptible. After recovering from COVID-19, individuals have temporary immunity in the short term and are not re-infected.
4. Different brands of vaccines have the same protective ability against novel coronaviruses.
5. A population-wide immune barrier has not yet been formed, and there are no effective drugs for treatment.
6. In the SEIR model, the “removed” compartments include both the recovered and deceased cases. In this study, our focus was primarily on trends in the maximum infection size, which is closely related to the healthcare burden. Therefore, we did not specifically address the number of deaths.

#### 2.2.2 Evaluation models

Using the susceptible, exposed, infectious and recovered (SEIR) compartmental model and associated parameters, a healthcare resource forecasting tool was developed for a scenario in which the risk of importation is predominant, and community transmission is still in its early stages. According to the scenario assumptions, the input parameters include: Susceptible (S), representing the general susceptibility of the population, i.e., the number of residents; Exposed (E), denoting the population that has been infected but has not yet shown symptoms of the disease. The initial number of this population is equal to *I*× σ ; Infectious (I), representing the population that has been infected and is in the onset of the disease. This population is related to the local case finding capacity and is greater than or equal to the number of confirmed cases. The initial size of this population can be reasonably assumed; Recovered (R), representing the population that has been cured or deceased.

The specific formula for the SEIR model is as follows (Equation 1):


$$\frac{dS}{dt}=-w\beta S\left(I+kE\right)/N$$

$$\frac{dE}{dt}=w\beta S\left(I+kE\right)/N-\sigma E$$

(1) $$\frac{dI}{dt}=\sigma E-\gamma I$$

$$\frac{dR}{dt}=\gamma I$$

N=S+E+I+R

**Figure 1:**
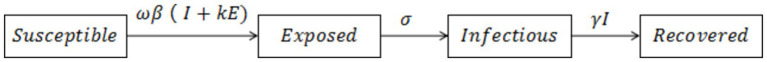
SEIR model propagation chain.

Figure 1 depicts the main infectious disease transmission structure of the model. The parameters used in the model are defined as follows: *β*: Coefficient of disease transmission, representing the average number of susceptible individuals infected by a single case of infection per unit of time, including both individuals who are ill and those in the incubation period. *σ*: Reciprocal of the incubation period, indicating the rate from infection to the onset of illness. *γ*: Reciprocal of the infectious period, indicating the rate of recovery from disease or death. *w*: Level of control intensity and the percentage of infections reduced by different levels of control measures. *k*: Coefficient of discounting infections between individuals infected during the latency period and those infected at the onset. Please refer to Table 2 for the values of other parameters.

**Table 2:** Variables associated with the SEIR model.

Parametric	Descriptions
S	Susceptible population (Total resident population)
*E*	Exposed (contagious but not showing symptoms)
*I*	Number of infected persons
*R*	Recovered^a^
w	Control intensity index, indicating the percentage of infections reduced by control measures at different risk levels
*β*	Transmission coefficient, indicating the average number of susceptible people who are infected by one infectious case (including those who are ill and those in the incubation period) in unit time
k	Infectivity discount coefficient of infected persons in incubation period compared with infected persons with onset
γ	Speed of recovery from disease
σ	The rate from infection to onset, namely the reciprocal of the incubation period
ν	Effectiveness of vaccination for infection prevention

a In the SEIR model, the compartment “removed” includes recovered and deceased cases. In this study, we focused more on the trends of the maximum infection scale, which is closely linked to the healthcare burden, and therefore did not include death cases.

**Figure 2:**
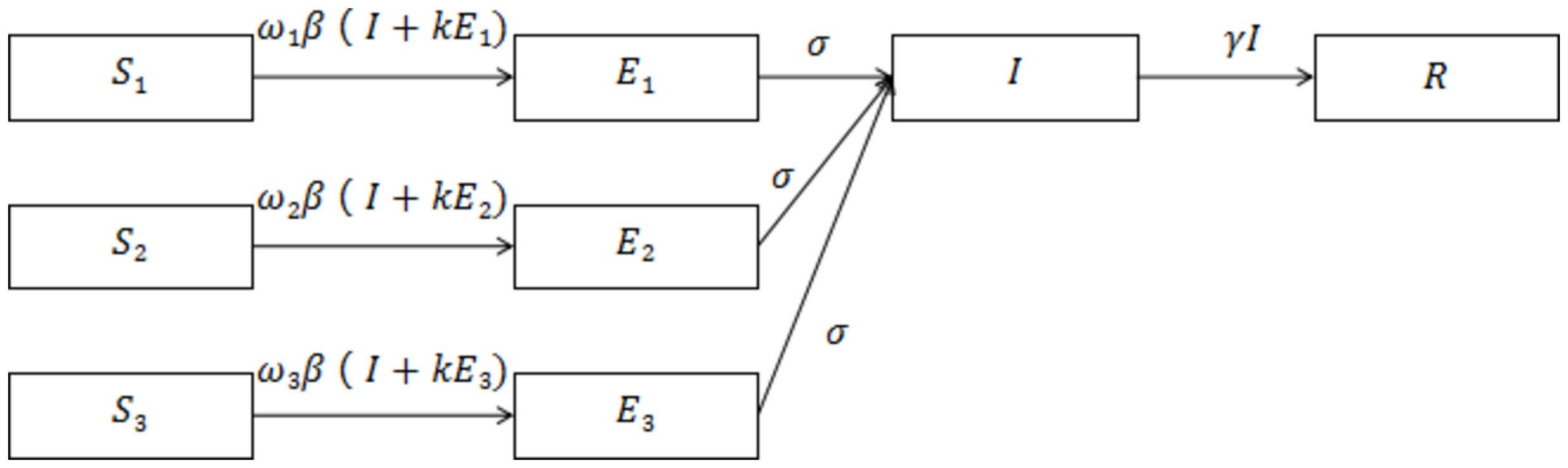
illustrates a model of the SEIR dynamic clearing propagation chain constructed based on the real-world implementation of the NPIs model. The model comprises eight compartments, where S1/E1 represents susceptible/exposed populations in high-risk areas, S2/E2 represents medium-risk areas, and S3/E3 represents low-risk areas. Cases with latency in other control areas are dynamically upgraded to high-risk areas when they develop symptoms after 3 days (t > 3).

Based on the SEIR model described above, we have further improved the model by incorporating a combination of measures for different levels of community control (Figure 2). This figure illustrates the transition of individuals between compartments based on disease state. Cases located in the incubation period of Level 1 (low-risk zones) and Level 2 (medium-risk zones) are dynamically promoted to Level 3 (high-risk zones) 3 days after the onset of symptoms (t > 3), as shown in Figure 2.

Different levels of control and modes of dissemination are factors that are taken into account in the model. Various levels of control measures can reduce the rate of exposure per unit of time. *ν* represents the effectiveness of vaccination, i.e., the percentage of the population vaccinated that is protected from infection. Based on the concepts developed for vaccine efficacy, the immune response produced by infection or vaccination reduces susceptibility to infection, decreases infectiousness, and mitigates pathology. All of these factors alter the values of the parameters in the model, and the system of differential equations is shown below (Equation 2).


$$\frac{dS_{1}}{dt}=-\frac{w_{1}\beta S_{1}\left(I+kE_{1}\right)}{N}$$

$$\frac{dS_{2}}{dt}=-\frac{w_{2}\beta S_{2}\left(I+kE_{2}\right)}{N}$$

$$\frac{dS_{3}}{dt}=-\frac{w_{3}\beta S_{3}\left(I+kE_{3}\right)}{N}$$

$$\frac{dE_{1}}{dt}=\frac{w_{1}\beta S_{1}\left(I+kE_{1}\right)}{N}-\sigma E_{1}$$

(2) $$\frac{dE_{2}}{dt}=\frac{w_{2}\beta S_{2}\left(I+kE_{2}\right)}{N}-\sigma E_{2}$$

$$\frac{dE_{3}}{dt}=\frac{w_{3}\beta S_{3}\left(I+kE_{3}\right)}{N}-\sigma E_{3}$$

$$\frac{dI}{dt}=\sigma \left(E_{1}+E_{2}+E_{3}\right)-\gamma I$$

$$\frac{dR}{dt}=\gamma I$$

$$N=S_{1}+S_{2}+S_{3}+E_{1}+E_{2}+E_{3}+I+R$$

#### 2.2.3 Description of variables and parameters

Three levels of NPI were included in this study. Based on our field research, the control measures implemented during the COVID-19 outbreak at levels 1, 2, and 3 reduced the total frequency of trips made by residents by 34, 54.3, and 88.9%, respectively. According to the formula $R_{0}=\beta *cˉ*d$ , i.e., the basic reproduction number = β * (probability of transmission per exposure) * (number of exposures per unit of time) * (period of transmission), three levels of NPI measures reduce the contact rate per unit of time, resulting in reductions in the effective number of regenerations (Rt) of 34, 54.3 and 88.9% (Table 3). The effectiveness of vaccination against COVID-19 was set at 33% after three doses of coronavirus vaccine, administered by intramuscular injection of 0.5 ml (3). Taking the three levels of control measures corresponding to the low-, medium- and high-risk zones as the baseline scenarios, and setting quantitative initial values based on the qualitative scenarios of population size, epidemic intensity, and effectiveness of prevention and control strategies and measures. The parameters for the virus were mainly adopted from the clinical progression parameters of COVID-19 in the literature, and the parameters for the effect of the control level were derived from field research during the epidemic control. The Omicron strain was only associated with hospitalizations due to its relatively mild symptoms of infection, and the hospitalization rate was calculated as 0.5% (4). The original strain of COVID-19 was associated with 13.8% severely ill patients and 4.7% critically ill patients (5). According to the local statistical yearbook, there are a total of 23,305 beds in the urban area of City A.

**Table 3:** Percentage reduction in total frequency of trips made by residents at different risk levels.

Form	Risk zones		
	Level 1 (low-risk zones)	Level 2 (medium-risk zones)	Level 3 (high-risk zones)
Percentage reduction in total frequency of trips	34.0%	54.3%	88.9%

#### 2.2.4 Scenario assumptions

Cities are the basic unit of healthcare resource allocation, and the scope of this study was chosen to represent a medium-sized city area. It was assumed that the resident population of an urban area in a prefecture-level city in Shandong Province is 2 million. Based on the epidemiological and virological characteristics of the infectious disease, a generally susceptible population in the city was assumed, and 10 different scenarios were constructed to simulate the epidemiological profile of the disease in a city with a population of 2 million people. Parameters, such as the epidemiological characterization of COVID-19 outbreaks, were obtained from previously published studies (6–9), expert professional opinions, and real-world surveys. Effective vaccines had not yet been developed by countries at the time of the epidemic of the original COVID-19 strain, so the effect of three doses of the coronavirus vaccine was included based on real-world considerations only in the simulation of the Omicron strain in Scenarios 1–5 (Table 4) and Scenario 9 (Table 5). The effect of the vaccine on the epidemic was not included in the simulation of Scenarios 6–8 (Table 6).

**Table 4:** Parameter combinations for five scenarios with different community control measures for Omicron strains.

Parameters	Scenario					Setting basis
	Scenario 1	Scenario 2	Scenario 3	Scenario 4	Scenario 5	
*S*	2 × 10^6^	2 × 10^6^	2 × 10^6^	2 × 10^6^	2 × 10^6^	Hypothesis
*E*	10	10	10	10	1750	I time the incubation period
*I*	3	3	3	3	500	Hypothesis
*R*	0	0	0	0	0	Hypothesis
w	–	34%	54.3%	88.9%	88.9%	Field research data
*β*	1.3 × 0.67	1.3 × 0.67	1.3 × 0.67	1.3 × 0.67	1.3 × 0.67	Reference (15, 16)
k	0.35	0.35	0.35	0.35	0.35	Reference (17)
γ	1/7	1/7	1/7	1/7	1/7	Reference (18)
σ	1/3.5	1/3.5	1/3.5	1/3.5	1/3.5	Reference (19)
ν	0.33	0.33	0.33	0.33	0.33	Reference (15)

**Table 5:** Initial parameter settings for Omicron strain scenarios with dynamic zeroing measures.

Parameters	Scenario 9	Setting basis
	Dynamic zeroing model for community control	
*S_1_*	0.25 × 10^6^	Hypothesis
*S_2_*	0.3 × 10^6^	Hypothesis
*S_3_*	1.45 × 10^6^	Hypothesis
*E_1_*	7	Hypothesis
*E_2_*	3	Hypothesis
*E_3_*	0	Hypothesis
*I*	33	Hypothesis
*R*	00	Hypothesis
w *_1_*	34%	Field research data
w *_2_*	54.3%	Field research data
w *_3_*	88.9%	Field research data
*β*	1.3 × 0.67	Reference (15, 16)
k	0.35	Reference (17)
γ	1/7	Reference (18)
σ	1/3.5	Reference (19)
ν	0.33	Reference (15)

**Table 6:** Parameter combinations for three scenarios of different community control measures COVID-19.

Parameters	Scenario			Setting basis
	Scenario 6	Scenario 7	Scenario 8	
*S*	2 × 10^6^	2 × 10^6^	2 × 10^6^	Hypothesis
*E*	10	10	10	I time the incubation period
*I*	3	3	3	Hypothesis
*R*	0	0	0	Hypothesis
w	–	54.3%	88.9%	Field research data
*β*	0.49	0.49	0.49	Reference (20)
k	0.35	0.35	0.35	Reference (17)
γ	1/14	1/14	1/14	Reference (10, 21, 22)
σ	1/3.2	1/3.2	1/3.2	Reference (20)
ν	-	-	-	

In the scenario assumptions, Scenarios 1–5 simulate the Omicron strain, while Scenarios 6–8 simulate the original strain of COVID-19 from the end of 2019 to the beginning of 2022, using different parameter combinations for each scenario. Specifically, the analysis is divided into the following steps:

Scenarios 1 and 6: These scenarios did not consider the effects of community NPIs and were intended to represent natural epidemiological scenarios used to exclude differences due to NPIs.

Comparison of Scenarios 2, 3, and 4 with Scenario 1: This comparison assessed the impact of different levels of NPIs (non-pharmaceutical interventions) on new coronavirus pneumonia. It involved describing and analyzing the timing of peak cases and the maximum number of cases.

Comparison of Scenarios 1–4 with Scenarios 6–8: This analysis focused on examining the impact of the same intensity of control measures on the prevalence trend of COVID-19 and Omicron strains in the same initial state.

Scenario 5: Describing the epidemiological trends that reflect the implementation of NPIs when the outbreak has reached the stage of widespread community transmission;

Comparison of Scenario 4 and Scenario 9: Analyzing the comparative effectiveness of implementing high-level NPI measures and dynamically discontinuing NPI measures in the same situation.

These scenario simulations allow for an in-depth understanding and assessment of outbreak trends under different parameter combinations, providing a scientific basis for the development of effective control measures. Please refer to Tables 4–7 for more detailed information on each scenario.

**Table 7:** Scenario settings.

Scenarios	Disease	NPI levels	Effectiveness of vaccination
Scenario 1	Omicron	No	33% for preventing infection
Scenario 2	Omicron	Level 1	33% for preventing infection
Scenario 3	Omicron	Level 2	33% for preventing infection
Scenario 4	Omicron	Level 3	33% for preventing infection
Scenario 5	Omicron	Level 3	33% for preventing infection
Scenario 6	COVID-19	No	-
Scenario 7	COVID-19	Level 2	-
Scenario 8	COVID-19	Level 3	-
Scenario 9	Omicron	Dynamic zeroing	33% for preventing infection

#### 2.2.5 Statistical analysis

The R software version 4.0.5 (Computational Foundations) and the deSolve package were used for modeling and analysis, while Microsoft Office 2016 was employed for data cleaning and description.

## 3 Results

### 3.1 The impact of NPIs on the early stages of the omicron epidemic—a comparison between Scenario 1, Scenario 2, Scenario 3, and Scenario 4

Scenario 1 Setting: Assuming a high level of case detection in an area and the initial detection of a major exotic respiratory infection. At this point in time, there were mainly imported cases, and the epidemic was still in its early stages. Considering factors such as the economic costs associated with control, no initial control measures were taken, i.e., the scenario was completely liberalized. The initial number of cases was 13, with 3 in the symptomatic phase (morbid phase) and 10 in the incubation phase. There were no deaths or recoveries reported at this stage (Table 4).

The Scenario 1 outbreak is projected to peak on Day 34 with a real-time incidence of 797,707. The peak number of hospitalized patients is 3,989. The number of new daily infections peaks at 229,547 on day 28, and the infection curve levels off after 91 days. The outbreak is expected to end after approximately 120 days. The results suggest that failure to implement preventive and control measures will lead to rapid and widespread community transmission. According to data from the Statistical Yearbook of City A, the total number of beds in the urban area is 23,305. Based on the available capacity of the existing inpatient beds in City A, although the beds are under pressure, they are still within manageable limits.

Scenario 2 assumes the implementation of only level 1 NPIs, resulting in a 34% reduction in transmission compared to Scenario 1. The other settings remain the same as in Scenario 1. The outbreak peaks on day 48, with a real-time incidence of 652,568. The peak number of hospitalized patients was 3,263, a decrease of 18.19% from Scenario 1, and the peak date was delayed by 14 days. The peak number of new infections per day was 145,758, a decrease of 36.5% from Scenario 1. After 128 days, the infection curve flattens out, and the outbreak lasts for approximately 140 days before coming to an end. The implementation of level 1 NPIs reduces the peak number of infections and hospitalizations and delays the peak, but still results in widespread transmission over a shorter period of time.

**Figure 3:**
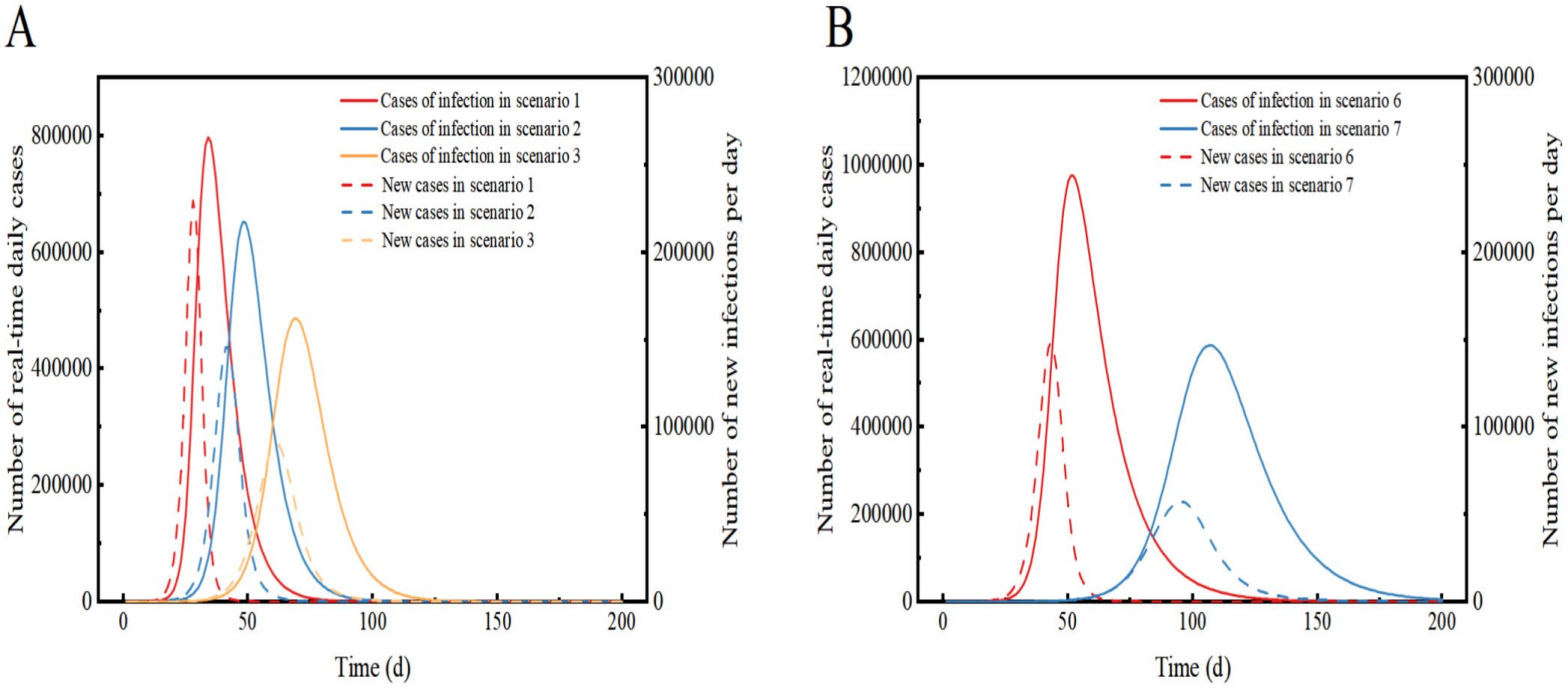
Comparison of the number of real-time daily cases and the number of new infections per day for Scenarios 1, 2 and 3 (A) and Scenarios 6 and 7 (B). In Scenarios 1, 2, and 3, the prevalent strain is assumed to be Omicron, while in Scenarios 6 and 7, the prevalent strain is assumed to be the original strain of COVID-19. The solid curve represents the number of real-time daily cases, and the dashed line represents the number of new infections per day.

**Figure 4:**
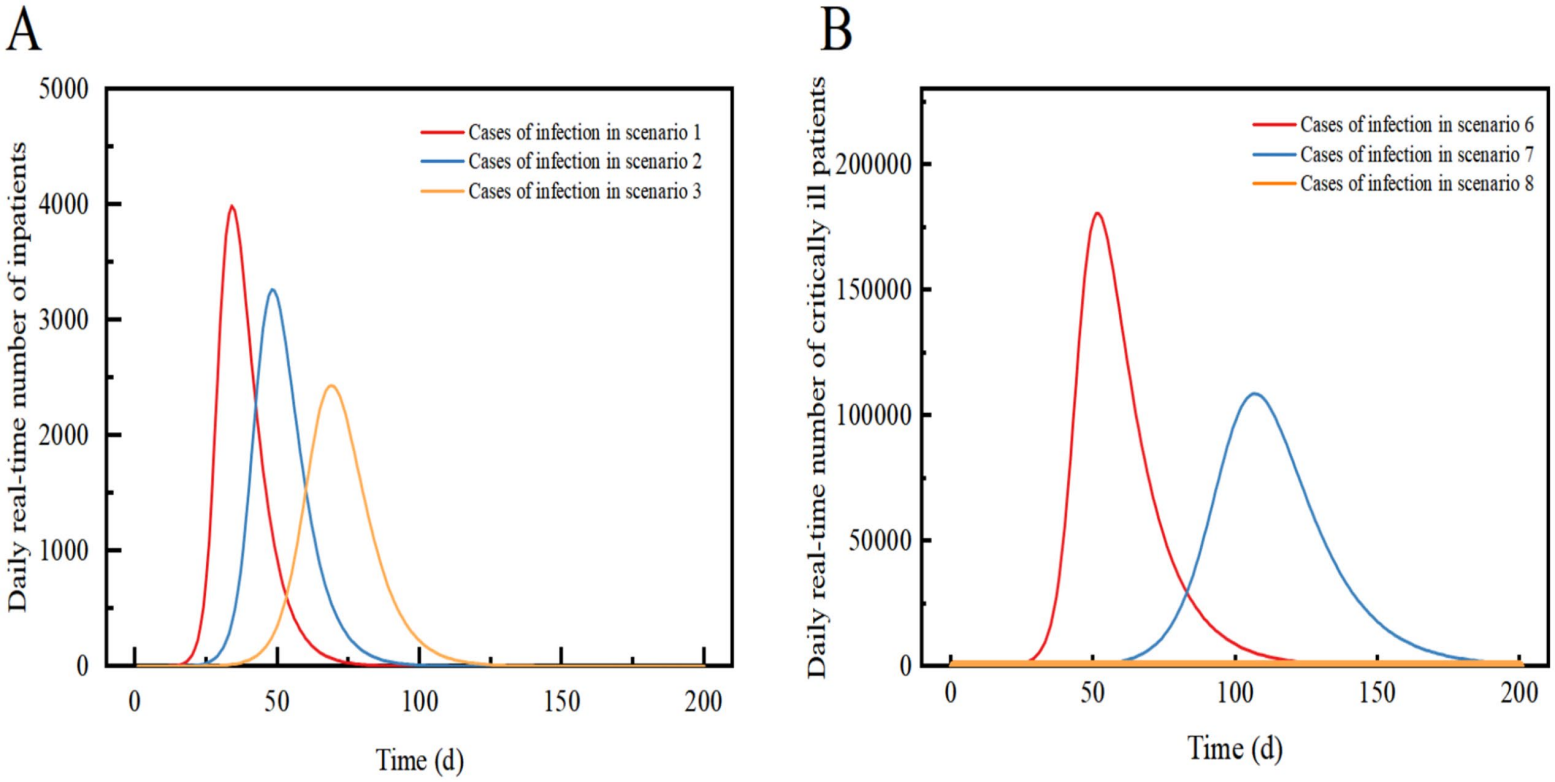
Comparison of the daily real-time number of inpatients in Scenario 1, Scenario 2, and Scenario 3 (A); Comparison of the daily real-time number of critically ill patients in Scenario 6, Scenario 7 and Scenario 8 (B). Scenarios 6, 7, and 8 simulate the early stages of an outbreak when an effective vaccine is not yet available. In Scenarios 1, 2, and 3, we assume that the proportion of vaccinated individuals preventing viral infections is 33%.

Scenario 3 assumes the implementation of level 2 NPIs, resulting in a 54.3% reduction in transmission compared to Scenario 1. The outbreak peaks on day 69 with 487,092 real-time cases, and the peak number of hospitalized patients of 2,435, a decrease of 38.94% from Scenario 1. The peak date is delayed by 35 days. The peak number of new infections per day was 90,500, a decrease of 60.57% compared to Scenario 1. After 167 days, the infection curve levelled off, and the outbreak ended after approximately 180 days. Level 2 NPIs significantly reduce the peak number of infections and hospitalizations, delay the peak, and decrease the demand for hospital beds. However, they also prolong the duration of the epidemic (Figures 3, 4A).

**Figure 5:**
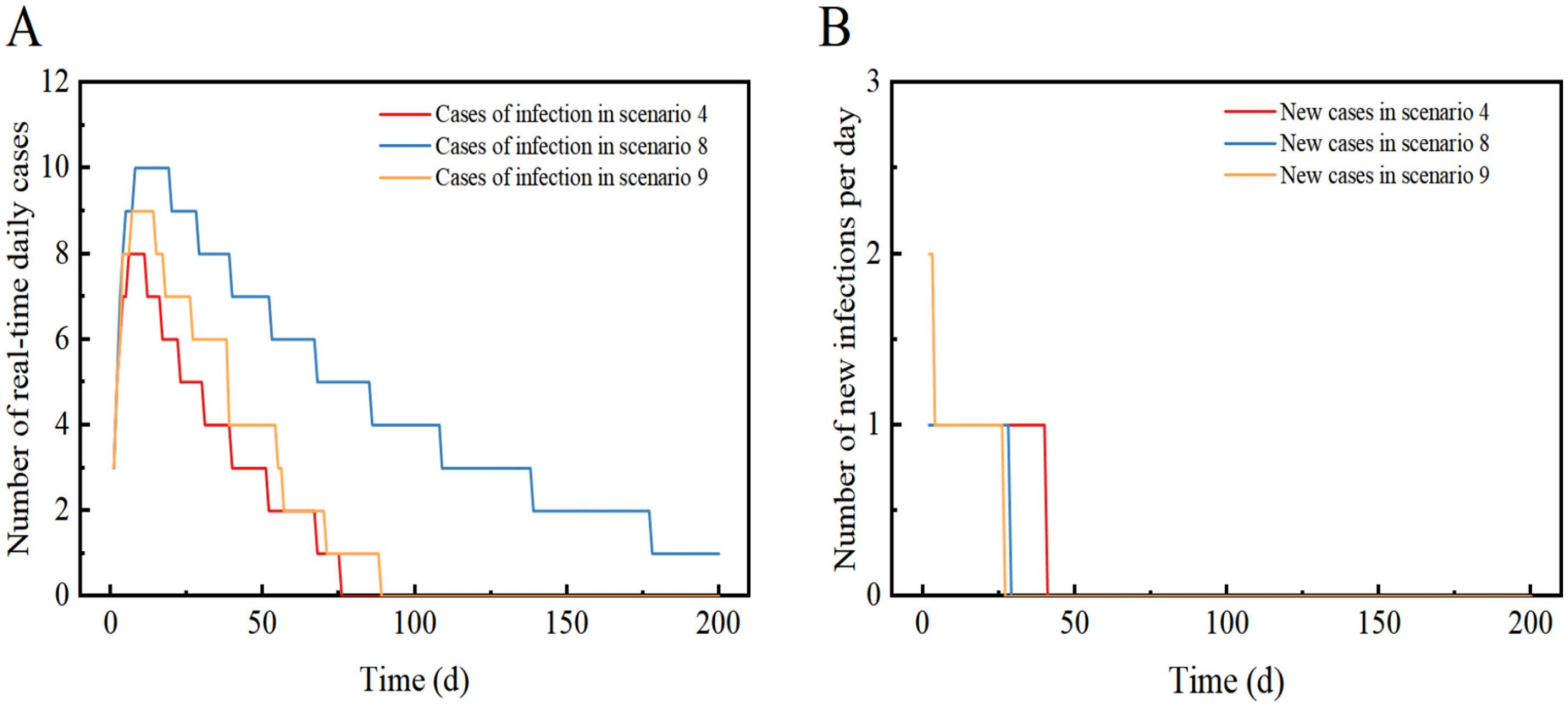
Scenarios 4, 8, and 9 show the change in the number of daily real-time cases in (A) and the number of new infections per day in (B). The scenario settings are displayed in Table 7. The parameter values used are listed in Tables 4–5.

Scenario 4 assumes the implementation of level 3 NPIs, resulting in an 88.9% reduction in transmission compared to Scenario 1. The outbreak peaks between days 5–11 with a maximum real-time incidence of only 8, which is 99.9% lower than in Scenario 1. After 40 days, the infection curve levels off, and the outbreak tends to end. Level 3 NPIs rapidly suppressed and extinguished the outbreak, preventing widespread community transmission and infection peaks (Figures 5A,B).

### 3.2 Impact of level 3 NPI on omicron epidemiology—Scenario 5

**Figure 6:**
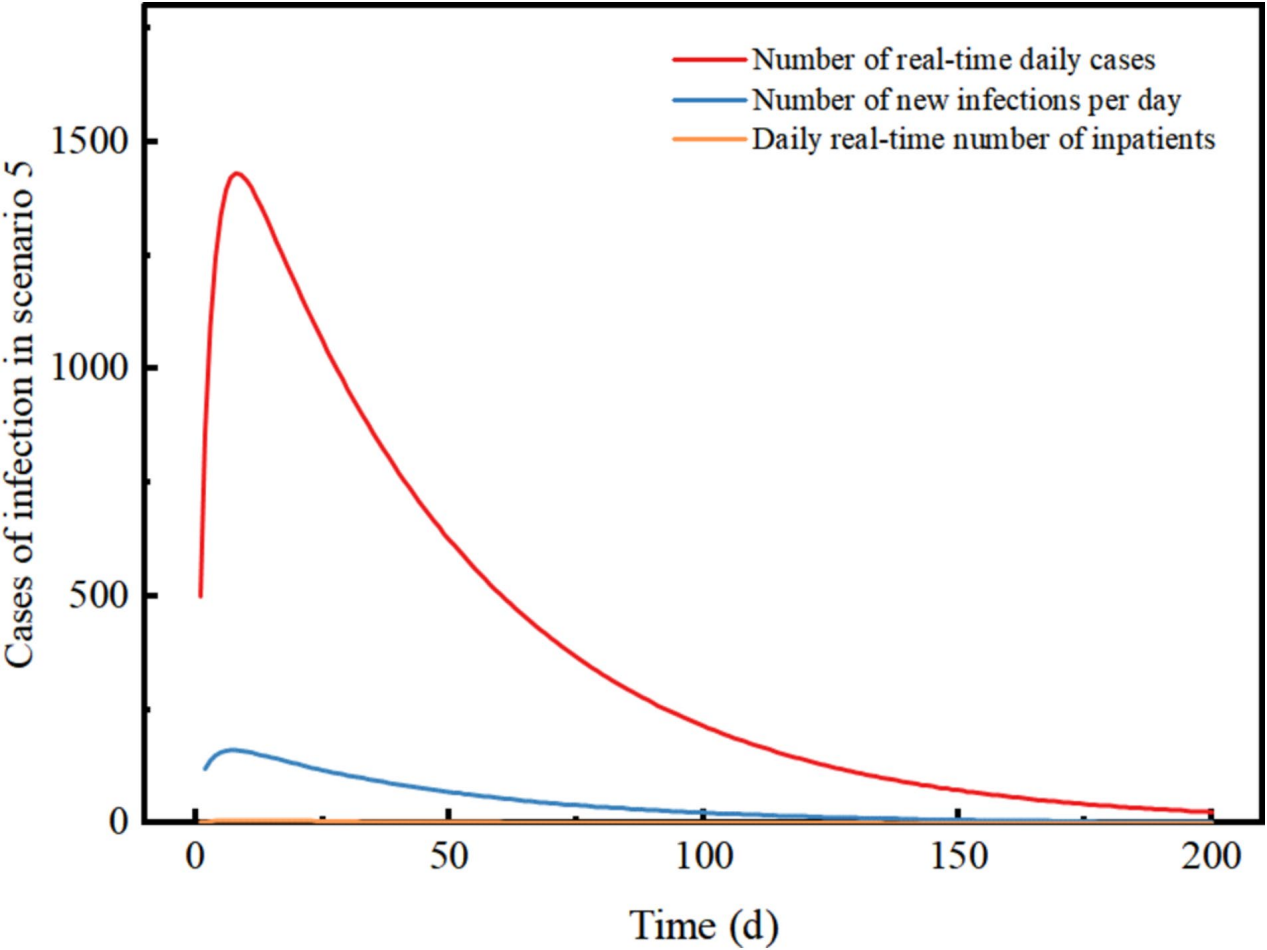
Changes in the number of different types of cases in scenario 5. The scenario settings are shown in Table 7. The parameter values used are shown in Table 4.

Scenario 5 assumes a late detection of the outbreak, with initially a high number of cases and widespread community transmission. After implementing level 3 NPIs, the control effect is 88.9%. The outbreak reaches its peak on day 8 and subsequently declines, with a peak real-time number of cases reaching 1,432. The peak number of inpatient admissions is 7, and on day 8, there are 162 new infections recorded. The epidemic lasted approximately 200 days. The results demonstrate that even with a high number of initial cases, the implementation of level 3 NPIs can swiftly control and eventually extinguish the outbreak. The need for hospitalization is low, but the duration of the epidemic is prolonged and comes with high socio-economic costs. Level 3 NPIs demonstrate a robust ability to decelerate the epidemic, and even with an increase in imported cases, the epidemic continues to show a downward trend (Figure 6).

### 3.3 Impact of NPIs on the early stages of the COVID-19 epidemic—comparison of Scenario 6, Scenario 7, and Scenario 8

Scenario 6 assumes that the epidemic strain is the original COVID-19 strain, with a high rate of severe illness, a slow recovery, mainly imported cases, and no NPIs implemented at the beginning of the epidemic. The epidemic peaks on day 52 with a real-time incidence of 975,757. At this point, the peak number of severely ill patients and hospital admissions exceeds those in Scenario 1 by 176,526, with the peak date delayed by 18 days. The peak number of new infections per day is 35.31% lower than in Scenario 1, with a peak date delayed by 15 days. The infection curve levels off after 156 days, and the epidemic ends after 200 days. The results suggest that the original strain of COVID-19 spreads more slowly than Omicron when NPIs are not implemented, leading to widespread community transmission after about a month. The slow recovery and high rate of severe illnesses result in a significant increase in the demand for healthcare resources, potentially causing a shortage of hospital beds in City A.

Scenario 7 assumes the same conditions as Scenario 6 but implements Level 2 NPIs with a 54.3% effect. The epidemic peaks on day 107 with 587,281 real-time cases, and the peak number of inpatient admissions is 39.81% lower than in scenario 6, with the peak date delayed by 55 days. The peak number of new infections per day is 61.33% lower than in scenario 6, with the peak date delayed by 52 days. The infection curve flattens out after approximately 200 days. The results shows that compared to a lack of NPI implementation, the peak number of infections and hospitalizations were significantly reduced, and the peak of the epidemic was significantly delayed. The demand for healthcare resources was alleviated, but there may still be a shortage of hospital beds in City A. When Level 2 NPIs were implemented, both the original strain of COVID-19 and Omicron showed similar suppression of real-time peak incidence. However, the original strain of COVID-19 was more sensitive to the delay in peak time and took longer to cause widespread community transmission (Figures 3, 4B).

Scenario 8 assumes the same conditions as scenario 6, but implements Level 3 NPIs with 88.9% effectiveness. The outbreak peaks on days 7–18, with a maximum real-time incidence of only 10, 99.9% lower than scenario 6. The infection curve flattens out after 29 days, but recovery is slow, and cases take a long time to clear. The results showed that Level 3 NPIs were effective in suppressing outbreaks and preventing widespread transmission. Compared to the Omicron strain in Scenario 4, Scenario 8 had a similar reduction in the peak number of infections, but it took longer to clear. Eventually, the outbreak was successfully stopped after slow spread without a peak infection (Figures 4B, 5A,B).

### 3.4 Impact of dynamic zeroing of NPI on the early stages of the omicron epidemic—Scenario 9

Scenario 9 assumes a high and more frequent level of case detection in the region, along with the detection of an imported case of a major respiratory infectious disease. In the early stages of the outbreak, three zones have been implemented to dynamically zero out NPIs. The initial number of imported cases is quantitatively set to 13, with 3 cases in the morbidity phase, 10 in the incubation phase, and no deaths or recoveries. Among these, 3 cases in the morbidity phase and 7 in the incubation phase are located in the Level 3 control area, while the remaining 3 in the incubation phase are in the Level 2 control area. The incubation period for three individuals in the Level 2 control area is set such that they develop symptoms of illness after 3 days (t > 3), following which they are dynamically upgraded to the Level 3 control area (Table 5).

Scenario 9 will peak between days 8–15 with a maximum real-time incidence of 9, similar to Scenario 4, suggesting that dynamic zeroing measures are close to the effectiveness of Level 3 NPIs. The infection curve levelled off after 27 days and the epidemic tended to end. This suggests that the implementation of dynamically cleared in only three zones can successfully interrupt the outbreak without a peak in infections (Figures 5A,B).

Overall, early detection and implementation of Tier 3 or dynamically cleared NPIs can be effective in controlling major respiratory infectious disease outbreaks by minimizing the peak number of infections and severe cases as well as reducing the duration of the outbreak.

## 4 Discussion

This study compares the course and magnitude of infection between the Omicron strain and the original COVID-19 strain under different scenarios. It provides a theoretical basis, modeling methodology, and assessment tools of general applicability. Additionally, it offers theoretical support and references for emergency preparedness of healthcare resources during a COVID-19 pandemic. Over the 3 years, the COVID-19 virus continued to mutate, with increased infectiousness and improved immune escape. However, the proportion of critically ill patients decreased. Critical cases have the greatest demand for healthcare resources, and we focused on analyzing the size of the critically ill population. To compare the scale of infection between Omicron variant and the original strain of COVID-19, we conducted modeling analyses based on the characteristics of the two strains. The research results show that during the March 22 epidemic in City A, the prevention and control effectiveness of Level 1 NPIs implemented in the precautionary zone (low-risk area) to hinder the spread of infectious diseases was 34%, the effectiveness of Level 2 NPIs in the control zone (medium-risk area) was 54.3%, and the effectiveness of Level 3 NPIs in the closure zone (high-risk area) was 88.9%, which is largely consistent with the findings of Zhang Ting (10) and other studies. The epidemics under scenarios with Level 1 or 2 NPIs all result in widespread community transmission, suggesting that only Level 3 community-based NPIs have a blocking effect on virus transmission. Assuming a scenario where most initial cases have already begun to spread within the community, even with the implementation of Level 3 NPIs, the outbreak would take approximately 200 days to be extinguished, necessitating a disproportionately high socio-economic cost. In China, as of July 20, 2022, 92% of the population had received at least one dose of COVID-19 vaccine, and 89% had been fully vaccinated with three doses. Consequently, we chose to set the parameters for vaccination after three doses of coronavirus vaccine (11). A study indicated that SARS-CoV-2 vaccination did not prevent the disease but did reduce its severity, transitioning cases from severe to mild or moderate (12–14). The high vaccination rate and stringent NPI helped maintain the COVID-19 outbreak in China at a low level.

### 4.1 The effectiveness of control measures is related to the level of intensity, timing, and characteristics of the virus

If Level 3 NPIs fail to stop the spread of the outbreak in the community, Level 1 and 2 NPIs can be implemented to reduce the peak daily incidence and postpone the peak’s arrival. Effective community NPIs and healthcare resource preparation can mitigate healthcare system overload and enhance affordability. The original Omicron and COVID-19 strains decreased in magnitude as community NPIs were elevated to higher levels. The findings of this study suggest that the implementation of community NPIs can significantly reduce the number of severe COVID-19 cases at the peak and delay the onset of the peak. Level 3 NPIs, in particular, can effectively reverse the course of an outbreak until it is extinguished. The use of high-level community NPIs is recommended to swiftly contain outbreaks when effective drugs and vaccines are not available. However, when the government decides to implement high-level community NPIs, it should also consider the economic costs, the psychological impact on community residents, and the potential increase in mortality due to limited access to healthcare services.

### 4.2 High-level and dynamically cleared NPIs can effectively prevent community transmission in specific contexts at the early stage of an epidemic

We observed that Level 3 NPIs significantly reduced epidemic infections in the short term. However, this measure is not cost-effective in the long term. A dynamic clearing model based on the SEIR model shows that before the viral transmission coefficient exceeds the blocking effect of Level 3 community NPIs, dynamic clearing is similar to the full implementation of Level 3 NPIs in the region, which aligns with China’s national conditions and balances epidemic prevention and economic development. With the mutation of COVID-19and the implementation of vaccination, several studies have indicated that the vaccine, while not halting the disease, has effectively reduced disease severity and viral pathogenicity. Due to the low readmission rate associated with Omicron, even in Scenario 1 without NPIs, the healthcare system in City A could potentially face a substantial but manageable shock, and the final scale of the outbreak would remain within the capacity of the healthcare system in many areas of China. When the predominant prevalent strain is the original COVID-19 strain, even with the full implementation of level 2 community NPIs within the city under the same Scenario 7, the healthcare system in City A could still collapse due to the peak in the number of individuals with severe/critical infections. The COVID-19 original strain is considered more dangerous not because it infects fewer people overall, but because, despite infecting fewer individuals than the Omicron variant, it has a higher rate of severe illness. This results in a relatively large number of severely ill patients, leading to a strain on healthcare resources in City A. This characteristic indicates that the original COVID-19 strain poses a greater danger than Omicron. Our model’s accurate reflection of the higher risk associated with the original strain when predicting or analyzing both strains is consistent with reality. In the late stages of the COVID-19 pandemic, the gradual relaxation of prevention and control policies by various countries further validates the model’s accuracy and effectiveness in assessing viral risk. Therefore, in order to effectively respond to a future severe respiratory pandemics, it will be crucial to ensure the orderly functioning of healthcare services through strict NPI measures and adequate healthcare resource preparedness. This represents the initial step in exploring how to enhance epidemic prevention and control measures.

## 5 Limitations

Scientific decisions should be based on sound evidence and epidemiological patterns. However, epidemics are complex, and it can be challenging to integrate all relevant indicators into models. First, when assessing the impact of NPI measures on infectious disease trends, we categorized the NPI measures into three intensity levels based on their risk but did not carefully classify or separately analyze the effects of each measure. Second, we referenced data on the effectiveness of three vaccine doses and assumed it to be constant, without accounting for the potential attenuation of effectiveness over time. Third, the study’s findings are based on generalized qualitative scenarios; therefore, if an assessment of health resource needs were to be carried out for cities or regions with larger populations, the quantitative initial values would need to be adjusted to the appropriate level. For example, since it is known that older people are more likely to be infected with COVID-19, the model could incorporate the age structure of the population using an age-stratified model, which could be explored in future studies. Finally, the data from the scenarios simulated using the model may not fully reflect the real situation.

## 6 Conclusion

In epidemics involving the original COVID-19 strain and Omicron, Level 3 community NPIs can extinguish outbreaks. Level 1 and 2 NPIs can slow down transmission, but medical collapse remains a risk with respiratory infections that have high rates of severe illness. Dynamic clearing can have a similar effect to the implementation of level 3 NPIs in the region. Therefore, the adoption of different levels of NPIs should be based on the severity rate of the virus, and sufficient medical resources should be prepared to preserve medical order.

## Data Availability

The raw data supporting the conclusions of this article will be made available by the authors, without undue reservation.
